# The complete chloroplast genome of *Zingiber striolatum* Diels (Zingiberaceae)

**DOI:** 10.1080/23802359.2022.2160218

**Published:** 2023-01-02

**Authors:** Shuming Tian, Dongzhu Jiang, Yuepeng Wan, Xiao Wang, Qinhong Liao, Qiang Li, Hong-Lei Li, Linzheng Liao

**Affiliations:** a College of Biology and Food Engineering, Chongqign Three Gorges University, Wanzhou, Chongqing, China; b College of Landscape Architecture and Life Science, Chongqing University of Arts and Sciences, Yongchuan, Chongqing, China; c College of Horticulture and Gardening, Yangtze University, Jingzhou, Hubei, China

**Keywords:** *Zingiber striolatum* Diels, chloroplast genome, phylogenetic analysis, genetic information

## Abstract

The chloroplast genome of *Zingiber striolatum* Diels was sequenced using the MGI paired-end sequencing method and assembled. The chloroplast genome was 163,711 bp in length, containing a large single-copy (LSC) region of 88,205 bp, a small single-copy (SSC) region of 15,750 bp, and two inverted repeat (IR) regions of 29,752 bp. The overall GC content was 36.1%, whereas the corresponding value in the IR regions was 41.1%, which was higher than that in the LSC region (33.8%) and SSC region (29.6%). A total of 136 complete genes were annotated in the chloroplast genome of *Z. striolatum*, including 87 protein-coding genes (79 protein-coding gene species), 40 tRNA genes (29 tRNA species), and 8 rRNA genes (4 rRNA species). A phylogenetic tree was constructed using the maximum likelihood (ML) method, and the results showed that the phylogeny of *Zingiber* was well resolved with high support values, and *Z. striolatum* was sister to *Z. mioga*. The assembly and sequence analysis of the chloroplast genome can provide a basis for developing high-resolution genetic makers.

## Introduction

*Zingiber striolatum* Diels (Diels 1901) is a perennial herb belonging to the genus *Zingiber* from the family Zingiberaceae (Tong 1998). *Z. striolatum* is a local vegetable, commonly known as Yang-He in China. The edible part of *Z. striolatum* is flowers (Huang et al. 2019). It is widely distributed, predominantly in many Chinese provinces with an altitude of about 800 m (An editorial committee of flora of China 1981; Huang et al. 2019). Although *Z. striolatum* could be cultivated with artificial plots in residential areas (e.g. in gardens), it is originally harvested from the wild. The plant height of *Z. striolatum* is 1–1.5 m (Deng et al. 2018). *Z. striolatum* has a cylindrical rhizome and an aromatic smell like other ginger species. *Z. striolatum* has high medicinal and edible value. The characteristics of *Zingiber* vegetative organs are highly similar, so it is extremely difficult to distinguish *Z. striolatum* from other *Zingiber* species (Theerakulpisut et al. 2012). DNA barcoding has been proved to be an automated, rapid, and accurate biological tool for species identification (Chen et al. 2010; Hebert et al. 2003). The informative sites of DNA barcoding markers are vital for the efficiency of species identification. Divergent hotspots for species identification have been reported in the subfamily Zingiberoideae (Li et al. 2021). However, the chloroplast genome of *Z. striolatum* has not yet been reported. In this study, we sequenced and assembled the complete chloroplast genome of *Z. striolatum*, which will contribute to understanding the population genetics of *Z. striolatum* and the chloroplast evolutionary dynamics of the genus *Zingiber*.

## Plant materials

**Figure 1.**
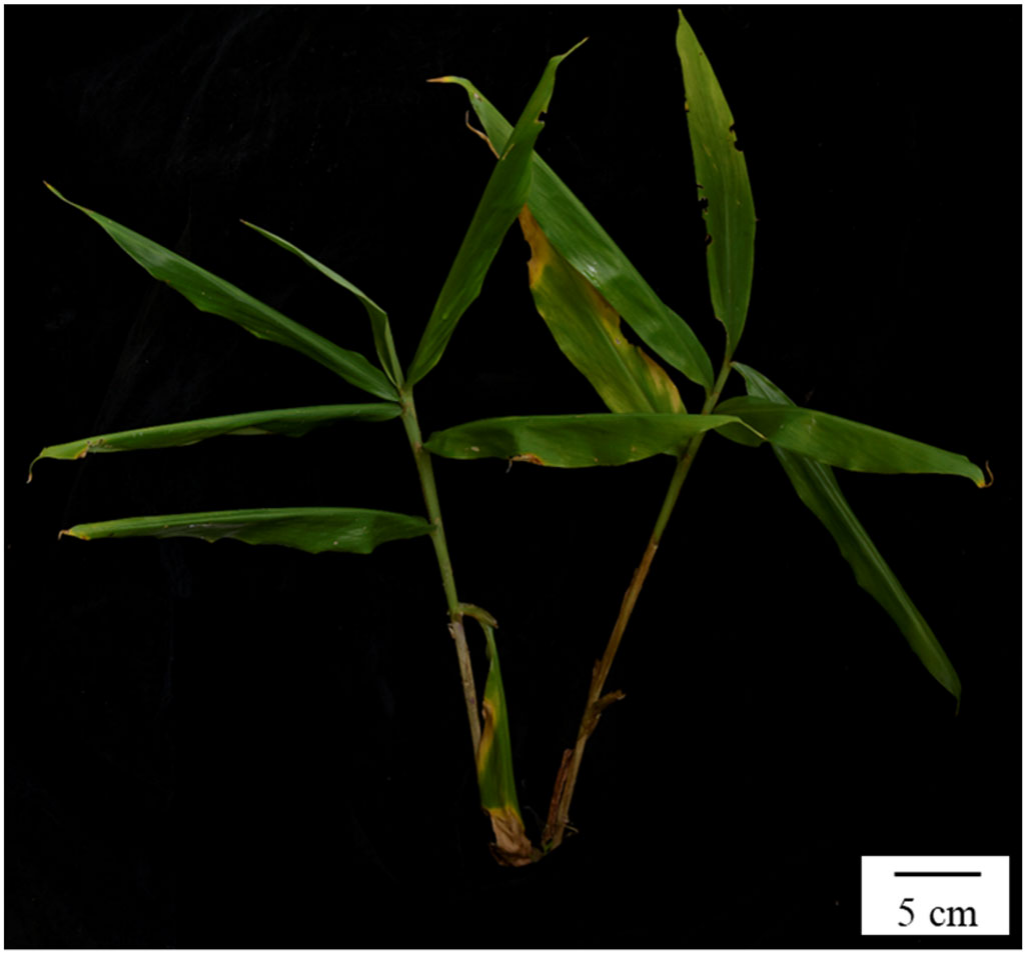
The aerial stem *of Zingiber striolatum* (photo taken by Hong-Lei Li took at Xishuangbanna Botanical Garden, Jinghong, Yunnan).

The plant materials of *Z. striolatum* sequenced in this study were collected from Xishuangbanna Botanical Garden, Jinghong City, Yunnan Province (21°41′N, 101°25′E) (Figure 1). This species is common in Southwest Yunnan, and no permission is required to collect these samples. Fresh and healthy leaves were dried with silica gel and preserved for DNA extraction. A specimen was deposited at the Herbarium of Chongqing University of Arts and Sciences (www.cqwu.net, Huamin Liu, liuhuanin@126.com) under the voucher number Li_Z033.

## Methods

The modified sucrose gradient centrifugation method was used to extract total genomic DNA from silica-gel-dried leaves (Li et al. 2012). The concentration and quantity of isolated genomic DNA were assessed using NanoDrop 2000 micro spectrometer (Wilmington, DE, USA). The isolated genomic DNA was stored in the −80 °C refrigerator of the Key Laboratory of the College of Landscape Architecture and Life Science. A paired-end DNA library with an insert size of 350 bp was prepared using the MGI Easy PCR-Free DNA Library Prep kit (MGI-TECH), and then was sequenced by the MGI DNBSEQ-T7 platform (MGI-TECH, ShenZhen, China). A total of 5.0 Gbp raw data were produced. To obtain clean data, the adaptor and low-quality sequences were removed. The software NOVOPlasty v3.5 was used to assemble the whole chloroplast genome. The software Genius v11.0.4 was used to annotate the chloroplast genome, the result of which was corrected using *Z. Officinal* (MW602894) as a reference genome.

The complete chloroplast genome sequences of *Z. striolatum* and 8 other *Zingiber* species were used to construct a phylogenetic tree, in which *Kaempferia galangal* was used as the outgroup. All the nine chloroplast genome sequences were aligned by MUSCLE with the default settings and refined manually. Then the ML tree was constructed using IQ-TREE with the default settings, including the GTRGAMMA model along with 1000 bootstrap (BS) replicates (Nguyen et al. 2015). All the above analyses were carried out using Geneious v11.0.4.

## Results

**Figure 2.**
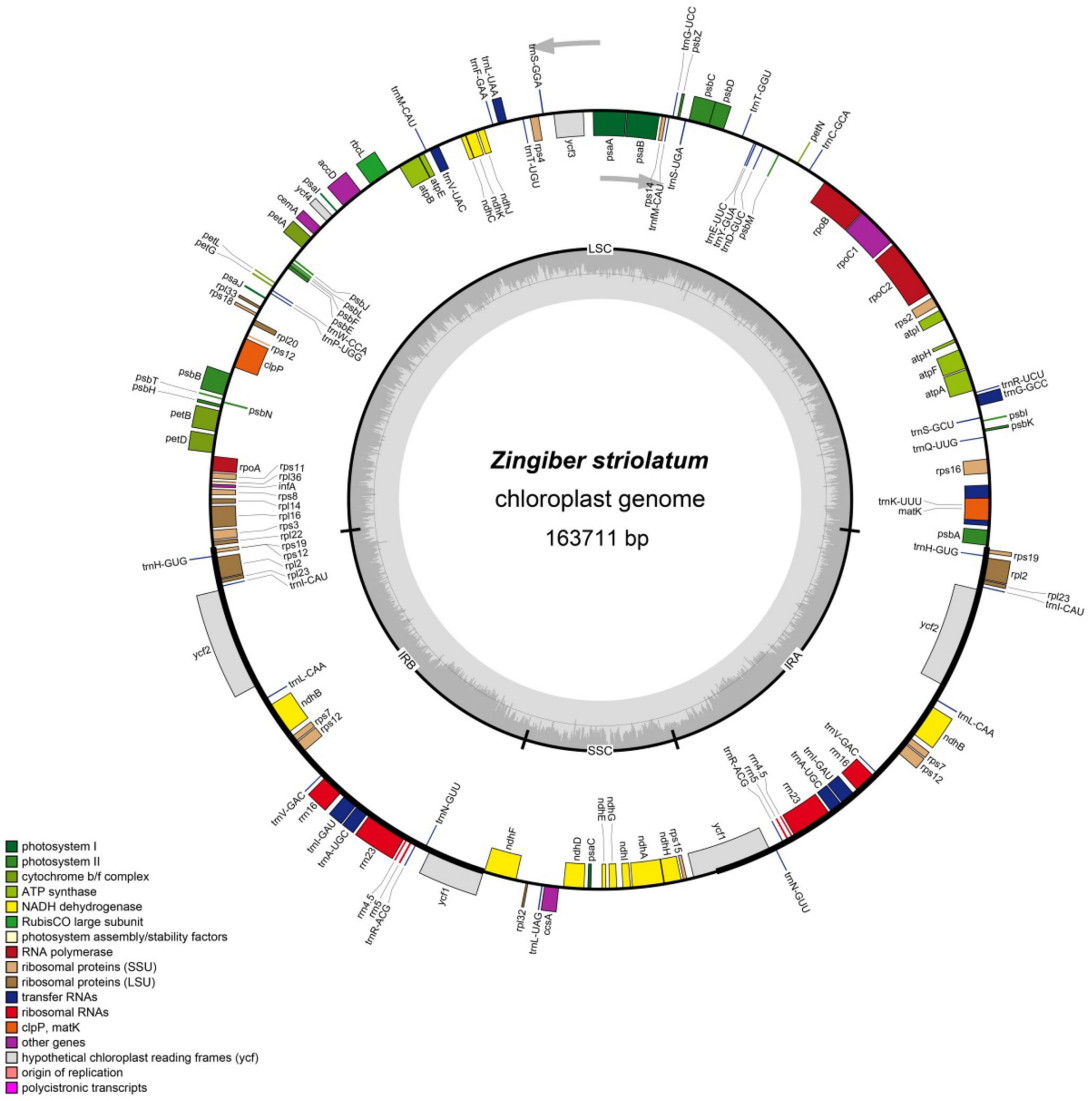
The structure of *Zingiber striolatum* chloroplast genome. The genes inside and outside of the circle are transcribed in the clockwise and counterclockwise directions, respectively. Genes belonging to different functional groups are shown in different colors. The darker gray area in the inner circle indicates the GC content and the lighter gray indicates the AT content of the chloroplast genome. The thick lines indicate the extent of the inverted repeats (IRA and IRB) that separate the chloroplast genome into the small single-copy (SSC) and large single-copy (LSC) regions.

The complete chloroplast genome of *Z. striolatum* (National Genomics Data Center accession number ON646165) was characterized from MGI paired-end sequencing. The chloroplast genome of *Z. striolatum* was 163,711 bp in length, containing one small single-copy (SSC) region of 15,750 bp, one large single-copy (LSC) region of 88,205 bp, and two inverted repeats (IRA and IRB) of 29,752 bp (Figure 2). The overall GC content was 36.1%, whereas the corresponding value in the IR regions was 41.1%, which was higher than that in the LSC region (33.8%) and the SSC region (29.6%). The sequenced chloroplast genome contained 136 predicted functional genes, including 87 protein-coding genes (79 protein-coding gene species), 40 tRNA genes (29 tRNA species), and 8 rRNA genes (4 rRNA species).

**Figure 3.**
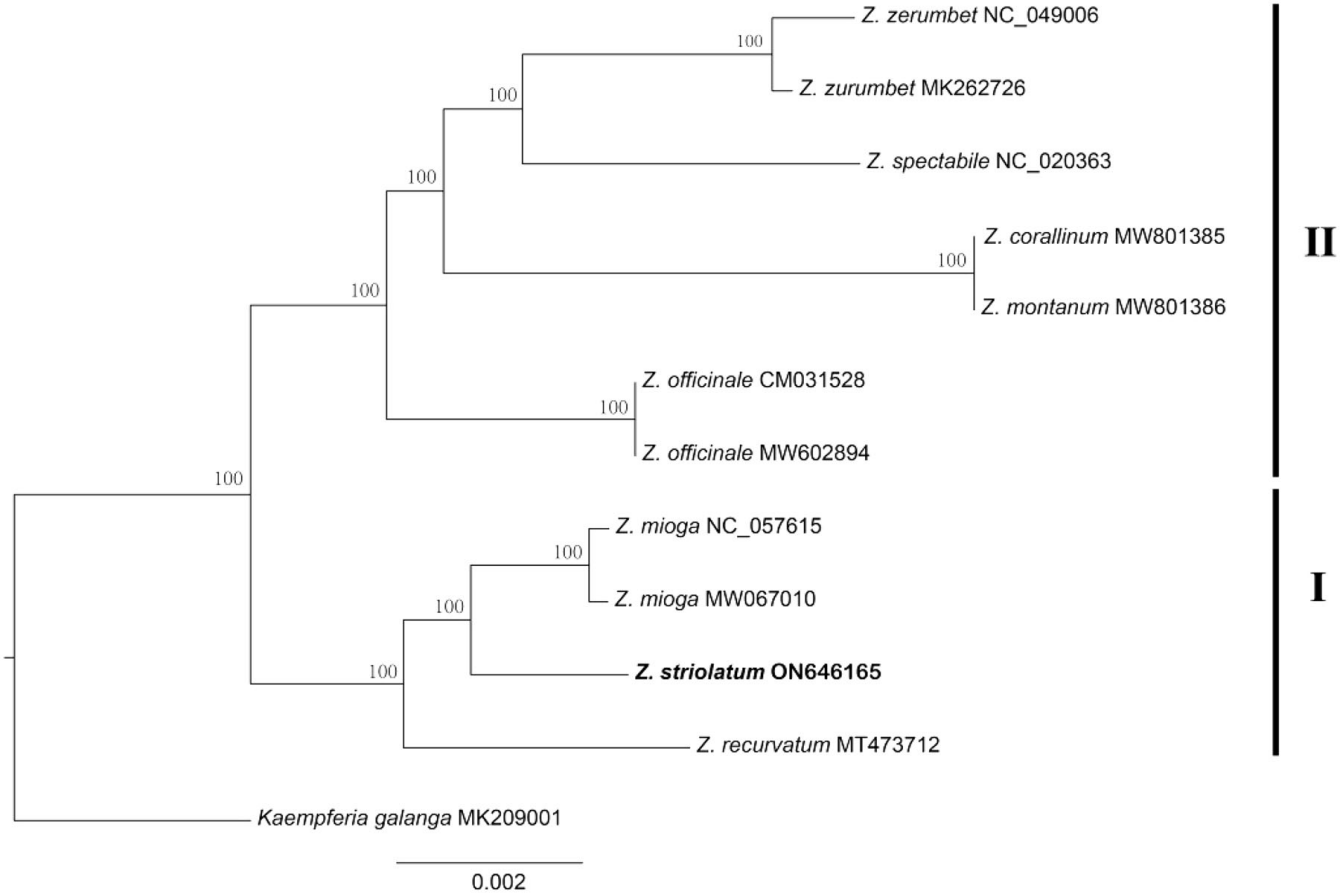
Phylogenetic tree of *Zingiber* inferred using maximum likelihood (ML) based on the complete chloroplast genome sequences. GenBank accession numbers: *Z. striolatum* (ON646165), *Z. recurvatum* (MT473712), *Z. mioga* (NC_057615), *Z. officinale* (MW602894), *Z. montanum* (MW801386), *Z. corallinum* (MW801385), *Z. spectabile* (NC_020363), *Z. zerumbet* (MK262726), and *Kaempferia gaalanga* (MK209001).

Based on the chloroplast genome dataset, we generated a well-resolved phylogenetic tree of *Zingiber* (Figure3). The support values of all the branches were robust (BS = 100%). Two well-supported lineages were recovered. Clade I contained *Z. recurvatum*, which was sister to *Z. mioga* and *Z. striolatum*. Clade II was composed of *Z. officinale* + ((*Z. corallinum* + *Z. montanum*) *+* (*Z. zerumbet* + *Z. spectabile*)).

## Discussion and conclusion

The genome size (163,711 bp), overall GC content (36.1%), genome quadripartite structure, and gene composition (including the protein-coding, tRNA, and rRNA genes) in the *Z. striolatum* chloroplast genome were highly similar to those in other *Zingiber* chloroplast genomes (Li et al. 2021). These results suggested that the chloroplast genomes of *Zingiber* species were highly conserved at the genus level. Overall, the phylogeny of *Zingiber* is congruent with the previous studies (Theerakulpisut et al. 2012; Li et al. 2021). Our phylogeny showed that *Z. recurvatum* was closely related to Z. *mioga*, which is consistent with the published molecular phylogeny based on chloroplast genome data (Li et al. 2021). Our results showed that *Z. zerumbet* was sister to *Z. spectabile*. Previous trees constructing using using either chloroplast genome sequences or nuclear ribosomal internal transcribed spacer (ITS) regions showed congruence of species relationship of Z. zerumbet and Z. spectabile (Theerakulpisut et al. 2012; Li et al. 2021). However, the support values of each branch within the phylogeny of *Zingiber* based on chloroplast genome data were higher than that based on nuclear genome data. These results indicated that the chloroplast genome is more effective in resolving the phylogenetic relationships among *Zingiber* species.

The assembly and sequence analysis of the chloroplast genome can provide a basis for developing high-resolution genetic makers, and also lay a foundation for subsequent studies on plant population genetics and genetic diversity.

## Data Availability

The genome sequence data that support the findings of this study are openly available in GenBank of NCBI at (https://www.ncbi.nlm.nih.gov/nuccore/ON646165) under the accession no. ON646165. The associated BioProject, SRA, and Bio-Sample numbers are PRJNA842832, SRR19426750, and SAMN28688325 respectively.
